# Influence of the Post-Harvest Storage Time on the Multi-Biological Potential, Phenolic and Pyrrolizidine Alkaloid Content of Comfrey (*Symphytum officinale* L.) Roots Collected from Different European Regions

**DOI:** 10.3390/plants10091825

**Published:** 2021-09-02

**Authors:** Adriana Trifan, Gokhan Zengin, Kouadio Ibrahime Sinan, Nils Esslinger, Andreas Grubelnik, Evelyn Wolfram, Krystyna Skalicka-Woźniak, Mirjana Minceva, Simon Vlad Luca

**Affiliations:** 1 Department of Pharmacognosy, “Grigore T. Popa” University of Medicine and Pharmacy Iasi, 700115 Iasi, Romania; adriana.trifan@umfiasi.ro; 2 Physiology and Biochemistry Research Laboratory, Department of Biology, Science Faculty, Selcuk University, Konya 42130, Turkey; sinankouadio@gmail.com; 3 Alpinamed AG, 9306 Freidorf, Switzerland; nils.esslinger@alpinamed.ch (N.E.); andreas.grubelnik@alpinamed.ch (A.G.); 4 Phytopharmacy and Natural Products Research Group, Zurich University of Applied Sciences, 8820 Wädenswil, Switzerland; wola@zhaw.ch; 5 Independent Laboratory of Natural Products Chemistry, Medical University of Lublin, 20-093 Lublin, Poland; kskalicka@pharmacognosy.org; 6 Biothermodynamics, TUM School of Life Sciences, Technical University of Munich, 85354 Freising, Germany; mirjana.minceva@tum.de

**Keywords:** *Symphytum*, storage, rosmarinic acid, globoidnan A, enzyme inhibition, antioxidant

## Abstract

Comfrey (*Symphytum officinale* L.) roots are well-known bioactive ingredients included in various cosmeceutical and pharmaceutical preparations. In this study, the influence of the post-harvest storage on the chemico-biological potential of roots collected from different European regions and stored for up to six months was investigated. Total phenolic content (TPC) and total phenolic acid content (TPAC) were spectrophotometrically estimated, whereas the levels of individual phenolic and pyrrolizidine alkaloidal markers were determined by HPLC-DAD and HPLC-MS/MS, respectively. The changes in the biological potential was tracked via antioxidant (DPPH, ABTS, CUPRAC, and FRAP) and anti-enzymatic (cholinesterase, tyrosinase, glucosidase, and amylase) assays. TPC and TPAC varied from 6.48–16.57 mg GAE/g d.w. root and from 2.67–9.03 mg CAE/g, respectively. The concentration of the four phenolics (rosmarinic acid, globoidnan A, globoidnan B, rabdosiin) and six pyrrolizidine alkaloids generally showed maximum values at 1–3 months, after which their levels significantly decreased. With respect to the bioassays, the samples showed a wide range of antioxidant and anti-enzymatic effects; however, a direct storage time–bioactivity relationship was not observed. Similar conclusions were also revealed by the multivariate and correlation analyses. Our study could improve the current knowledge of the shelf-life properties of comfrey-based products and enhance their industrial exploitation.

## 1. Introduction

The Eurasian genus *Symphytum* (Boraginaceae) comprises around 40 perennial species, such as *S. officinale* L. (comfrey), *S.* × *uplandicum* Nyman (Russian comfrey), *S. asperum* Lepech (prickly comfrey), *S. tuberosum* L., S. *anatolicum* Boiss, *S. aintabicum* Hub.-Mor. & Wickens, and *S. caucasicum* Bieb [1,2,3,4,5]. External (ointments, compresses) and internal (infusions, tinctures) preparations obtained from aerial parts (*Symphyti herba*), leaves (*Symphyti folium*), and, especially, roots (*Symphyti radix*), have been empirically used since Ancient times as traditional remedies in bruises, burns, phlebitis, tonsillitis, and respiratory, gastrointestinal, and urinary ailments [6,7].

Previous in vitro and in vivo studies have already reported a plethora of biological activities of extracts obtained from different *Symphytum* species (i.e., antimicrobial, antioxidant, anti-inflammatory, wound-healing, anti-nociceptive, anti-enzymatic) [3,4,5,7,8,9,10]. Furthermore, numerous randomized clinical trials have shown that topical comfrey formulations are able to ameliorate the pain, inflammation, and swelling of muscles and joints from arthritis, bone fractures, contusions, or sprains [11,12].

Comfrey roots are conventionally acknowledged to contain polysaccharides (i.e., mucilage, 29%), purine derivatives (i.e., allantoin, 0.6–4.7%), amino acids, glycopeptides, phenolic acids, triterpene saponins, and pyrrolizidine alkaloids (0.013–1.2%) [6]. Comfrey polysaccharides are reported to exert antioxidant, anticancer, immunomodulatory, hypoglycemic, and hypolipidemic properties [13], whereas allantoin possesses wound-healing (extracellular matrix synthesis, fibroblastic proliferation) and immunomodulatory effects [2]. As a well-known constituent of comfrey roots, rosmarinic acid is endowed with antioxidant, antimicrobial, anticancer, and anti-inflammatory activities [7,10]. In addition, three oligomeric lignans (i.e., globoidnan A, globoidnan B, and rabdosiin) have been previously isolated by our group and confirmed as major phenolic markers in commercial comfrey root batches [10,14]. Pyrrolizidine alkaloids (i.e., intermedine, lycopsamine, acetylintermedine, acetyllycopsamine, symphytine) are a large group of 1,2-unsaturated necine ring structures that can occur either as free bases or as *N*-oxides. Due to their geno-, cyto-, pneumo-, and hepato-toxicity, the therapeutic applications of comfrey preparations are partly overshadowed [7,9]. For instance, the Committee on Herbal Medicinal Products (HMPC) of the European Medicines Agency (EMA) allowed the use of comfrey cutaneous preparations only on intact skin and in doses lower than 1 μg/day [15].

To the best of our knowledge, there is currently no information about the influence of post-harvest storage time on the chemico-biological potential of comfrey. To investigate this, roots of *S. officinale* were collected from five different European regions, stored for up to six months, and periodically analyzed. The variability of the phytochemical profile was assessed with respect to total phenolic content (TPC) and total phenolic acid content (TPAC), as well as to the levels of individual phenolic and pyrrolizidine alkaloidal markers. To track the influence of storage on the biological potential of comfrey roots, a complex battery of in vitro antioxidant (radical scavenging, reducing power, and metal chelating) and anti-enzymatic (anti-cholinesterase, anti-tyrosinase, anti-glucosidase, and anti-amylase) bioassays were performed. Lastly, the chemico-biological differences related to the geographical region and post-harvest storage time were approached from an exploratory multivariate perspective.

## 2. Results

### 2.1. Quantitative Assessment of Total Phenolics, Total Phenolic Acids, and Individual Phenolic Markers in Comfrey (S. officinale) Roots

In this work, five comfrey root samples (coded as *FS*, *IS*, *NT*, *CH,* and *PL*) were collected from different European regions (Table S1) and stored for 1–6 months. TPC and TPAC were assessed spectrophotometrically, whereas the concentration of the major phenolic markers (rosmarinic acid, globoidnan A, globoidnan B, and rabdosiin) were determined by high performance liquid chromatography hyphenated with diode array detection (HPLC-DAD). Overall, the levels of all measured parameters varied in relation to storage period and source (Table 1). Sample *NT* displayed the highest TPC at M1 and M3 (16.57 and 15.47 mg gallic acid equivalents (GAE)/g (d.w. root), respectively), whereas the lowest TPC was observed in sample *FS* at M6 (6.48 mg GAE/g). On the other hand, TPAC varied from 2.67 mg caffeic acid equivalents (CAE)/g (*FS* at M6) to 9.03 mg CAE/g (*NT* at M1). From the first to the sixth month of storage, a significant decrease by 28%, 59%, and 10% of TPAC was noticed in samples *FS*, *NT,* and *CH*, respectively.

Generally, samples *CH* (3.50–4.98 mg/g) and *NT* (2.82–5.05 mg/g) exhibited the highest rosmarinic acid levels (Table 1). Rosmarinic acid decreased after 6 months of storage by 30–47% in all five samples, in comparison to the first month. The second individual phenolic marker monitored in this study, globoidnan A, reached the maximum amount in sample *NT* at M2 (3.60 mg/g) and the lowest value in *PL* at M2 (0.78 mg/g). After six months, its levels were reduced by 19% (*PL*)–60% (*NT*) in comparison to the first month of storage. Globally, sample *CH* displayed the highest content of globoidnan B (1.53–2.50 mg/g); its amounts at M6 decreased by 50%, 25%, and 39% in samples *FS*, *IS,* and *CH*, respectively. Lastly, rabdosiin levels varied from 0.14 mg/g (*FS* at M6) to 1.26 mg/g (*CH* at M1). Considering the entire storage period, rabdosiin content followed the same trend noticed for the previous three phenolic markers, with levels diminished by 29% (*PL*), 42% (*IS*), 44% (*CH*), 51% (*NT*), and 63% (*FS*) at M6 vs. M1.

### 2.2. Quantitative Assessment of Pyrrolizidine Alkaloids in Comfrey (S. officinale) Roots

The main comfrey pyrrolizidine alkaloids were quantified by HPLC hyphenated with tandem mass spectrometry (HPLC-MS/MS). Thus, four individual constituents (intermedine, lycopsamine, intermedine-*N*-oxide, and lycopsamine-*N*-oxide) and two stereoisomeric pairs (acetylintermedine+acetyllycopsamine and acetylintermedine-*N*-oxide+acetyllycopsamine-N-oxide) were monitored (Table 2). Intermedine and lycopsamine were not quantifiable (N.q.) in samples *FS*, *IS*, *NT,* and *PL*, whereas very low levels (0.01 mg/g of each) were found in sample *CH*. From all five samples, *CH* also contained the highest amounts of acetylintermedine+acetyllycopsamine (0.08–0.14 mg/g), whilst the values in the other samples did not exceed 0.03 mg/g. Intermedine-*N*-oxide attained the maximum concentrations (0.23–0.36 mg/g) in sample *IS*. In contrast, its stereoisomer, lycopsamine-*N*-oxide generally reached the highest values in sample *FS* (0.22–0.50 mg/g). Interestingly, the levels of both pyrrolizidine alkaloids significantly decreased after a 6-month storage period, with the most dramatic decreases for lycopsamine-*N*-oxide (by 93%) and intermedine-*N*-oxide (by 56%) in sample *FS*. Acetylintermedine-*N*-oxide+acetyllycopsamine-*N*-oxide was the most abundant pyrrolizidine alkaloid group, with amounts ranging from 0.49 mg/g (*NT* at M6) to 4.26 mg/g (*FS* at M1). The storage period also showed a significant impact on the levels of these alkaloids, especially after the sixth month, with significant reductions by 37% (*IS*), 40% (*PL*), 41% (*CH*), 53% (*NT*), and 61% (*FS*) as compared to the first month.

### 2.3. Assessment of the Antioxidant Activity of Comfrey (S. officinale) Roots

To investigate the influence of the post-harvest storage period on the bioactivity of comfrey roots, the antioxidant potential was further assessed. Radical scavenging activity was evaluated by 1,1-diphenyl-2-picrylhydrazyl (DPPH) and 2,2′-azino-bis(3-ethylbenzothiazoline) 6-sulfonic acid (ABTS) assays; metal reducing and chelating activity by cupric ion reducing antioxidant capacity (CUPRAC), ferric ion reducing antioxidant power (FRAP), and ferrous ion chelating ability (MCA) assays; and total antioxidant capacity by a phosphomolybdenum assay (PDA).

Sample *NT* displayed the highest DPPH and ABTS radical scavenging properties, especially at M1 (38.15 and 157.76 mg Trolox equivalents (TE)/g in DPPH and ABTS assays, respectively) and M3 (38.49 and 173.25 mg TE/g in DPPH and ABTS assays, respectively). However, at M6, the radical scavenging activity decreased by 44% and 47% in the two tests, as compared to M1 (Table 3). Interestingly, sample *FS* showed a 2.5–5-fold increase in DPPH and ABTS radical scavenging activity at M3 followed by a significant decrease in activity at M6. Nevertheless, for samples *IS* and *PL*, the anti-radical effects were almost constant, regardless of the 6-month storage period, whereas sample *CH* exhibited the most dramatic changes at M6, when the activity decreased by 73–81%, as compared to M1.

A similar trend was noticed in the two performed reducing assays. For instance, sample *NT* showed superior CUPRAC (80.13 mg TE/g) and FRAP (90.67 mg TE/g) at M1, in comparison to the other samples. For samples *NT* and *CH,* the cupric and ferric ion reducing properties significantly decreased (2–2.5-fold) after the sixth month, whereas the related bioactivities of samples *IS* and *CH* suffered very small changes over the investigated storage period (Table 3).

With respect to MCA, sample *IS* displayed the highest activity (5.90–7.61 mg EDTA equivalents (EDTAE)/g). Generally, the maximum MCA was attained at M1–M3, with significant reductions by 22% in sample *IS*, 26% in sample *NT,* and 87% in sample *CH* at M6 (Table 3). Lastly, total antioxidant activity reached the highest potential in samples *NT* at M1 (0.79 mmol TE/g) and *PL* at M6 (0.79 mmol TE/g). In samples *NT* and *CH*, PDA revealed a 1.5–2.6-fold activity reduction over the whole storage period, whilst the total antioxidant capacity of the remaining samples showed small or no variations at M6 vs. M1 (Table 3).

### 2.4. Assessment of the Enzyme Inhibitory Activity of Comfrey (S. officinale) Roots

To complement the influence of the post-harvest storage period on the bioactivity, the enzyme inhibitory properties of comfrey roots were subsequently evaluated.

The two anti-cholinesterase assays revealed lack of acetylcholinesterase (AChE) inhibitory activity for samples *FS* and *IS* (Table 4). Interestingly, sample *CH* was inactive at M1–M3, but it showed good anti-AChE properties (3.32 mg galanthamine equivalents (GALAE)/g) at M6. Samples *NT* and *PL* were found active over the whole period storage, with the maximum AChE inhibitory potential at M3 for sample *NT* (4.21 mg GALAE/g) and M6 for sample *PL* (4.08 mg GALAE/g). Overall, the anti-butyrylcholinesterase (BChE) activity of comfrey roots decreased to a very small extent at M6 as compared to M1, with sample *NT* suffering the most dramatic reduction (by 36%).

For sample *CH*, tyrosinase inhibitory potential achieved its maximum at M6 (18.08 mg kojic acid equivalent (KAE)/g), whereas for samples *PL* (18.05 mg KAE/g), *NT* (13.80 mg KAE/g), *IS* (7.17 mg KAE/g), and *FS* (6.92 mg KAE/g), the highest anti-tyrosinase activity was observed at M3.

The amylase inhibitory effects varied from 0.03 mg acarbose equivalents (ACAE)/g (*FS* at M6) to 0.16 mg ACAE/g (*CH* at M1), whereas considerably higher anti-glucosidase properties were noticed, from 0.21 mg ACAE/g (*FS* at M6) to 0.51 mg ACAE/g (*NT* at M3 and *PL* at M6). Except for sample *PL*, the inhibitory activities against the two anti-diabetic enzymes significantly decreased after the sixth month of storage, as compared to the first month. For instance, activity reductions of 3.3-fold and 1.8-fold were noticed in sample *FS* with respect to the anti-amylase potential and sample *NT* with respect to the anti-glucosidase potential, respectively (Table 4).

### 2.5. Exploratory Multivariate and Correlation Analyses

To obtain further insights, the influence of the post-harvest storage period and geographical region on the chemico-biological potential of comfrey roots was investigated from a multivariate perspective. Principal component analysis (PCA) was firstly used to determine any homogeneous group of samples with common characteristics.

**Figure 1:**
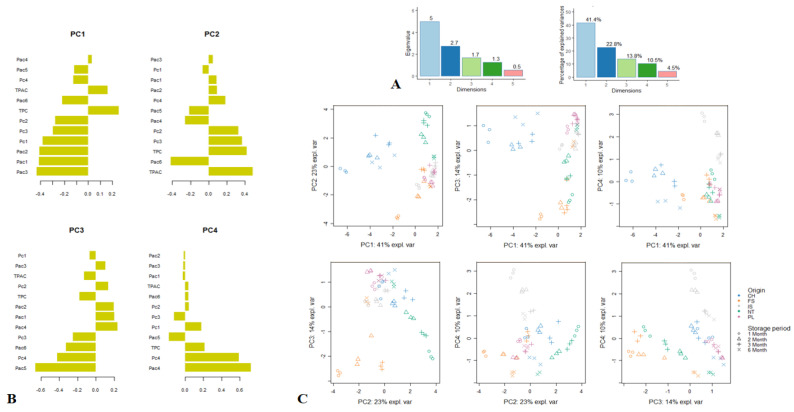
Principal component analysis (PCA) showing the variability of the phytochemical composition of comfrey roots depending on storage period and geographical region. (**A**). Eigenvalues and percentage of explained variance of each principal component (PC). (**B**). Loading plots displaying the relationship between the phytochemicals and the four significant PCs. (**C**). Score plots showing the distribution of the samples in the six two-dimensional plans obtained from the four significant PCs; Pac1, intermedine; Pac2, lycopsamine; Pac3, acetylintermedine/acetyllycopsamine; Pac4, intermedine-*N*-oxide; Pac5, lycopsamine-*N*-oxide; Pac6, acetylintermedine-*N*-oxide/acetyllycopsamine-*N*-oxide Pc1, rosmarinic acid; Pc2, globoidnan A; Pc3, globoidnan B; Pc4, rabdosiin; TPAC, total phenolic acid content; TPC, total phenolic content.

The PCA of the phytochemical profile data manifested a cumulative variance of 88.5% (PC1 = 41.4%, PC2 = 22.8%, PC3 = 13.8%, PC4 = 10.5%) (Figure 1A). Loading plots were graphed to probe the relationship between phytochemical compounds and the four retained principal components (PCs) (Figure 1B). These PCs were retained since they gave eigenvalues greater than 1.0. PC1 discriminated the samples mainly according to their content in intermedine (Pac1), lycopsamine (Pac2), acetylintermedine/acetyllycopsamine (Pac3), and rosmarinic acid (Pc1). PC2 separated the samples predominantly based on their amounts of TPC, TPAC, and acetylintermedine-*N*-oxide/acetyllycopsamine-*N*-oxide (Pac6). PC3 partitioned the samples principally in terms of their levels of lycopsamine-*N*-oxide (Pac5) and rabdosiin (Pc4), while PC4 differentiated the samples based on their content in intermedine-*N*-oxide (Pac4) and rabdosiin (Pc4). Subsequently, the score plots representing the positioning of the samples in comparison to each other were shown in Figure 1C.

**Figure 2:**
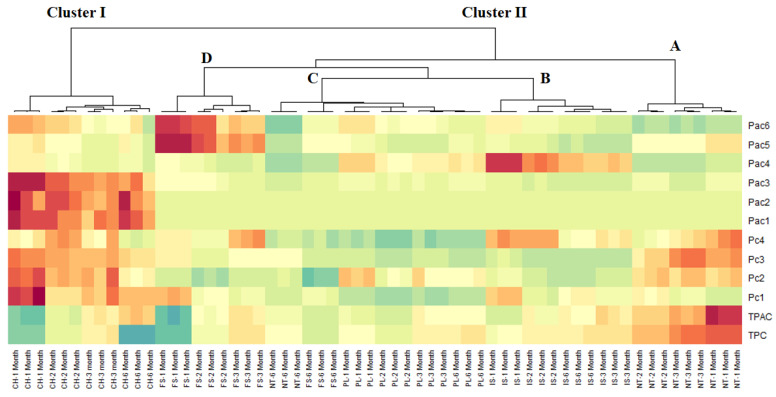
Cluster image map (CIM) analysis of the phytochemical composition data of comfrey roots (red color: high concentration, blue color: low concentration). Pac1, intermedine; Pac2, lycopsamine; Pac3, acetylintermedine/acetyllycopsamine; Pac4, intermedine-*N*-oxide; Pac5, lycopsamine-*N*-oxide; Pac6, acetylintermedine-*N*-oxide/acetyllycopsamine-*N*-oxide Pc1, rosmarinic acid; Pc2, globoidnan A; Pc3, globoidnan B; Pc4, rabdosiin; TPAC, total phenolic acid content; TPC, total phenolic content.

Given the fact that the analysis of the score plots did not allow the facile identification of different homogeneous groups, cluster image map (CIM) analysis was next performed. CIM analysis revealed a better visualization of the different groups of samples; thus, two main clusters (with one divided into four sub-clusters) were obtained (Figure 2). Cluster I (samples *CH* at M1–M6) was richer in numerous constituents, such as intermedine (Pac1), lycopsamine (Pac2), acetylintermedine/acetyllycopsamine (Pac3), rosmarinic acid (Pc1), globoidnan A (Pc2), and globoidnan B (Pc3). Cluster IID (samples *FS* at M1–M3) was particularly characterized by its high content in lycopsamine-*N*-oxide (Pac5) and acetylintermedine-*N*-oxide/acetyllycopsamine-*N*-oxide (Pac6), whereas cluster IIB (samples *IS* at M1–M6 months) was richer in intermedine-*N*-oxide (Pac4).

**Figure 3:**
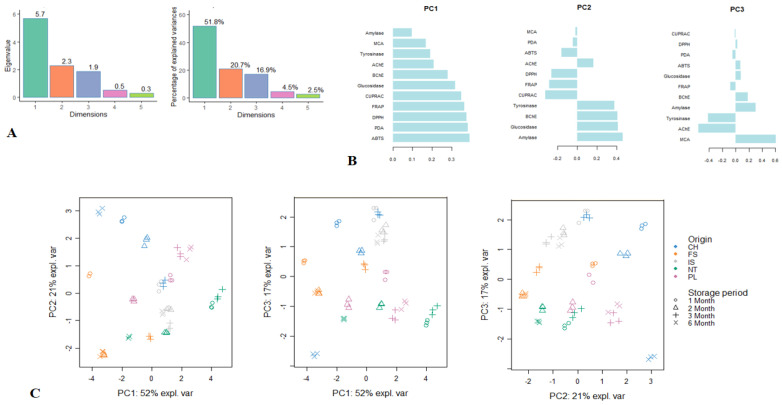
Principal component analysis (PCA) showing the variability of the antioxidant and anti-enzymatic activity of comfrey roots depending on the storage period and geographical region. (**A**). Eigenvalues and percentage of explained variance of each principal component (PC). (**B**). Loading plots displaying the relationship between the biological activities and the three significant PCs. (**C**). Score plots showing the distribution of the samples in the three two-dimensional plans obtained from the three significant PCs. ABTS, 2,2′-azino-bis(3-ethylbenzothiazoline) 6-sulfonic acid; AChE, acetylcholinesterase; BChE, butyrylcholinesterase; CUPRAC, cupric ion reducing antioxidant capacity; DPPH, 1,1-diphenyl-2-picrylhydrazyl; FRAP, ferric ion reducing antioxidant power; MCA, metal chelating activity; PDA, phosphomolybdenum activity.

Next, PCA of the biological activity data was performed and presented in Figure 3. Three significant PCs accounting for 89.4% of the total variance were distinguished for the analysed data (Figure 3A). PC1 accounted for 51.8% of the variance, and it was predominantly linked to the antioxidant activity. PC2 summarized 20.7% of the variance, and it was principally characterized by amylase, glucosidase, BChE, and tyrosinase inhibitory activities. Accounting for 16.9% of the variance, PC3 was determined by the high loading of MCA and anti-AChE activity. Loading plots (Figure 3B) were graphed to show the links between bioactivities and the three retained PCs. Figure 3C shows the repartition of the samples in the two-dimensional plan formed by the three components.

**Figure 4:**
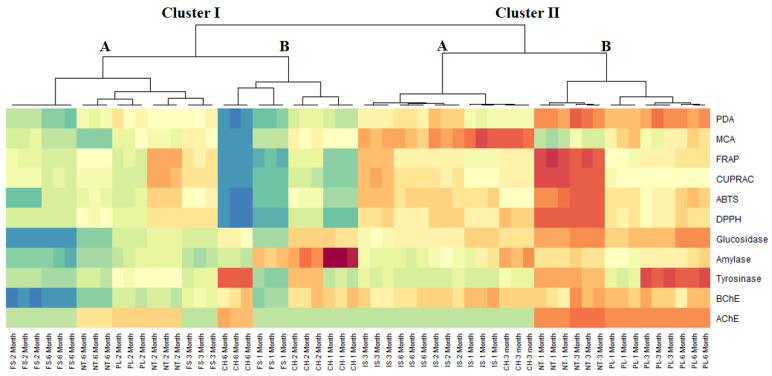
Cluster image map (CIM) analysis of the antioxidant and anti-enzymatic activity data of comfrey roots (red color: high activity, blue color: low activity). ABTS, 2,2′-azino-bis(3-ethylbenzothiazoline) 6-sulfonic acid; AChE, acetylcholinesterase; BChE, butyrylcholinesterase; CUPRAC, cupric ion reducing antioxidant capacity; DPPH, 1,1-diphenyl-2-picrylhydrazyl; FRAP, ferric ion reducing antioxidant power; MCA, metal chelating activity; PDA, phosphomolybdenum activity.

Due to the great variability between the samples, identifying different groups by PCA was quite challenging. The implementation of CIM analysis, starting with the coordinates of each samples on the three PCs of PCA, revealed two major clusters, each divided in two sub-clusters (Figure 4). Remarkably, samples belonging to cluster II, especially those of sub-cluster IIB (*NT* at M1 and M3 and *PL* at M1, M3, and M6), exhibited the strongest biological activities.

**Figure 5:**
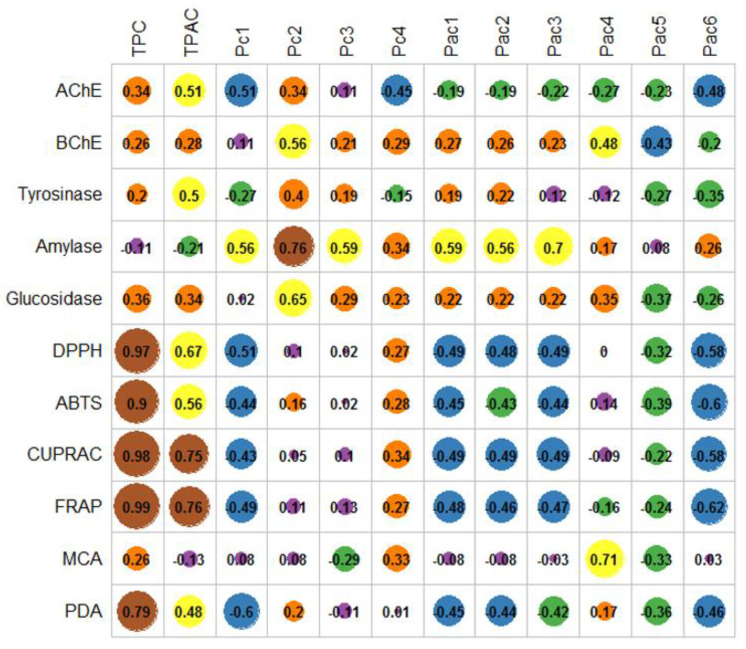
Correlation analysis between the phytochemical composition and biological activities. ABTS, 2,2′-azino-bis(3-ethylbenzothiazoline) 6-sulfonic acid; AChE, acetylcholinesterase; BChE, butyrylcholinesterase; CUPRAC, cupric ion reducing antioxidant capacity; DPPH, 1,1-diphenyl-2-picrylhydrazyl; FRAP, ferric ion reducing antioxidant power; MCA, metal chelating activity; Pac1, intermedine; Pac2, lyocopsamine; Pac3, acetylintermedine/acetyllycopsamine; Pac4, intermedine-*N*-oxide; Pac5, lycopsamine-*N*-oxide; Pac6, acetylintermedine-*N*-oxide/acetyllycopsamine-*N*-oxide; Pc1, rosmarinic acid; Pc2, globoidnan A; Pc3, Globoidnan B; Pc4, rabdosiin; PDA, phosphomolybdenum activity; TPAC, total phenolic acid content; TPC, total phenolic content.

Subsequent to the exploratory multivariate analysis, the correlation analysis between the biological activity and phytochemical composition was performed and depicted in Figure 5. A significant relationship was found between antioxidant activity and TPC. In addition, only CUPRAC and FRAP were correlated to TPAC, whereas MCA was linked to rabdosiin (Pc4). Regarding the anti-enzymatic activities, only anti-amylase activity was significantly correlated with globoidnan A (Pc2) and acetylintermedine/acetyllycopsamine (Pac3). Furthermore, a moderate relationship (Pearson’s coefficients > 0.5) was observed between some phytochemicals and radical scavenging, anti-glucosidase, anti-amylase, and anticholinesterase activities (Figure 5).

## 3. Discussion

Comfrey roots (*Symphyti radix*) have been traditionally used since ancient times, mostly for their analgesic and anti-inflammatory properties [6,16]. Recent studies that relied on modern analytical and preparative chromatographic platforms (i.e., liquid-chromatography hyphenated with high-resolution tandem mass spectrometry (LC-HRMS/MS), liquid–liquid chromatography (LLC)) have shown that comfrey roots are an underestimated reservoir of biomolecules. For instance, preceding phytochemical experiments have revealed previously unmapped constituents, notably globoidnan A, globoidnan B, rabdosiin, comfreyn A, caffeic acid ethyl ester, α-hydroxyhydrocaffeic acid, ternifoliuslignan D, 3-carboxy-6,7-dihydroxy-1-(30,40-dihydroxyphenyl)-naphthalene, and various oxygenated fatty acids [10,17,18]. With the aim to expand the medical values of the genus beyond its current uses, various research groups have demonstrated the antioxidant, neurobiological, hypoglycemic, hypolipidemic, or pro-osteogenic potential of various *Symphytum* species [3,4,5,9,13,19].

Even though the ethnopharmacological studies have shown that various formulations (tinctures, ointments, compresses, and decocts) are *ex tempore* prepared from fresh roots, generally collected from March to June and from September to October [1,8], dried comfrey roots are commercially available (i.e., in pharmacies, herbal stores) and further processed domestically (in household) or industrially (large-scale) [16]. However, to the best of our knowledge, there are no previous studies to address the influence of the post-harvest storage time on the chemico-biological potential of *S. officinale* L.

In this study, the variability of the phytochemical profile of comfrey roots collected from different European regions, dried, and stored for up to six months was evaluated with respect to TPC, TPAC, and the content of four individual phenolic and six pyrrolizidine alkaloidal markers. On the other hand, to assess the impact on the biological potential, in vitro antioxidant (radical scavenging, metal reducing, and metal chelating) and anti-enzymatic (anti-AChE, anti-BChE, anti-tyrosinase, anti-glucosidase, and anti-amylase) activities were investigated. This particular panel of bioassays constitutes a starting point in the sinuous road of discovering novel leading candidates in the management of pathological conditions associated with oxidative stress, such as Alzheimer’s disease, skin pigmentation disorders, or diabetes [20].

When compared to one-month storage (M1), TPC and TPAC generally decreased by 10–48% and 10–59%, respectively, in comfrey roots analyzed after six months (M6). Nevertheless, samples at M2 and/or M3 often displayed the highest TPC and TPAC values. Rosmarinic acid, globoidnan A, globoidnan B, and rabdosiin were identified, no matter the geographical coordinates of the collection sites. However, inter- and intra-sample variability was clearly noticed. For instance, sample *NT* reached the highest levels of rosmarinic acid (5.05 mg/g) and globoidnan A (3.60 mg/g), whereas sample CH exhibited the highest concentrations of globoidnan B (2.50 mg/g) and rabdosiin (1.26 mg/g). Interestingly, the previous analysis of the same phenolic markers in 16 commercial comfrey root batches acquired from different European countries displayed lower levels (max. 1.94 mg rosmarinic acid/g, max. 1.93 mg globoidnan A/g, max. 0.99 mg globoidnan B/g, and max. 0.88 mg rabdosiin/g) [14]. With respect to the storage time, our results showed that the individual phenolic constituents reached the highest values at M1–M3, after which a significant reduction (by 19–60%) became noticeable at M6. The current phytochemical data could imply that post-harvest metabolic activation reactions, well-described in numerous fresh fruits and vegetables [21,22,23], might still occur in dried comfrey materials for a certain period of time (up to 2–3 months), before enzymatic or physico-chemical degradative processes become prevalent (up to six months). Previously, it has only been shown that the harvest time can have a significant influence on the phytochemical composition of fresh comfrey roots. The levels of rosmarinic acid in materials collected in the first and second year of cultivation did not suffer major changes, whereas the concentration of globoidnan A decreased dramatically from one year to the other [2].

Raising serious safety concerns for the human health, EMA highly recommends assessing the levels of pyrrolizidine alkaloids in nutraceutical, cosmeceutical, and pharmaceutical products prior to commercialization. In the current study, intermedine and lycopsamine were found in amounts lower than 0.01 mg/g, whereas the concentration of their *N*-oxide derivatives was below 0.50 mg/g. From the two pairs of stereoisomers (acetylintermedine+acetyllycopsamine and acetylintermedine-*N*-oxide+acetyllycopsamine-*N*-oxide), the first one reached maximum levels of 0.14 mg/g, whereas the latter one achieved unexpectedly high values (between 0.49 and 4.26 mg/g). Our results are comparable to those reported for the commercial root batches, when slightly higher levels for intermedine (max. 0.10 mg/g), lycopsamine (max. 0.11 mg/g), intermedine-*N*-oxide (max. 1.69 mg/g), and lycopsamine-*N*-oxide (max. 1.87 mg/g) and lower levels of acetylintermedine+acetyllycopsamine (max. 0.12 mg/g) and acetylintermedine-*N*-oxide+acetyllycopsamine-*N*-oxide (max. 2.67 mg/g) were noticed [14].

Furthermore, the post-harvest storage time showed a significant impact on the stability of the pyrrolizidine alkaloids (their concentration generally decreased in a time-dependent manner from M1 to M6). This could suggest an improvement in the safety profile of comfrey roots after storage. However, considerably lower action levels (below 1 μg/day) are still imposed by EMA [15]. Nevertheless, genetically modified cultivars that do not biosynthesize pyrrolizidine alkaloids or different techniques to selectively deplete the materials are highly researched and applied at pilot or industrial scales [24,25].

To investigate the influence of the post-harvest storage time on the biological potential, antioxidant and anti-enzymatic assays were conceptually performed. With respect to inter-sample variability, it was noticed that sample *NT* exhibited overall the highest CUPRAC, FRAP, DPPH, and ABTS radical scavenging properties, whereas sample *IS* showed superior metal-chelating effects. The total antioxidant activity was at a maximum in the first three months of storage, after which it stayed at a baseline level (i.e., in samples *IS* and *CH*) or decreased by 1.5–2.6 fold in the remaining samples. There were no considerable inter- and intra-sample changes in the anticholinesterase activity of comfrey roots, whilst the tyrosinase inhibitory effects showed no direct storage time–activity correlations. The anti-glucosidase activity (0.21–0.51 mg ACAE/g) of the investigated samples was superior to their anti-amylase activity (0.03–0.16 mg ACAE/g). A similar situation was also observed in a previous report, when the glucosidase inhibition of aerial part and root extracts of *S. officinale* was 25–50 times stronger than the amylase inhibition [9].

In contrast to the phytochemical analysis that revealed considerable reductions of TPC, TPAC and levels of phenolic and pyrrolizidine alkaloidal markers, the overall bioactivity (antioxidant and anti-enzymatic) of comfrey roots stored for up to six months did not display remarkable time-dependent changes. These outcomes were reinforced by the correlation analysis between the biological activity and chemical composition, when only moderate Pearson’s coefficients between several groups of phytoconstituents and radical scavenging, anti-glucosidase, anti-amylase and anticholinesterase were noticed. In a previous study [9], 66 different minor and major specialized metabolites were annotated by LC-HRMS/MS in different polarity solvents (dichloromethane, methanol, and 65% ethanol) of aerial parts and roots of *S. officinale*. From these, several individual phenolic acids, such as danshensu, dihydrogloboidnan B, rabdosiin, rosmarinic acid, and dihydrogloboidnan A, were positively correlated in DPPH, ABTS, CUPRAC, FRAP, and total antioxidant capacity assays. In addition, no remarkable relationships between phenolic acids and the anti-enzymatic activity have been noticed. However, positive correlations between sucrose (indicative for the presence of polysaccharides) and the anti-AChE, anti-BChE, anti-tyrosinase, anti-amylase, and anti-glucosidase potential have been noticed. Intermedine-*N*-oxide, 7-acetylintermedine-*N*-oxide and 7-acetyllycopsamine-*N*-oxide, sarracinyl-9-trachelantylretronecine, 7-sarracinyl-9-viridiflorylretronecine, symphytine-*N*-oxide, and symlandine-*N*-oxide were linked with anti-AChE, anti-tyrosinase, and anti-glucosidase activity [9].

## 4. Materials and Methods

### 4.1. Plant Material and Extraction

Comfrey (*Symphytum officinale* L.) roots from multiple plants (around 39 specimens) were collected in September–October 2020 from five different geographical regions (Table S1) and authenticated by one of the authors (A.T). Within each geographical region, the plant samples were obtained from similar populations and pooled together. Voucher specimens (Table S1) were deposited in the Department of Pharmacognosy, *Grigore T. Popa* University of Medicine and Pharmacy Iasi (Romania). The roots were dried for one month in an acclimatized room (20 ± 2 °C; 55 ± 5 relative humidity). After grinding, the powdered materials were stored for 6 months in the dark in brown flasks under the same acclimatized room conditions. Extractions were performed from the homogenized powdered material at 1 month, 2 months, 3 months, and 6 months after the collection time. The extractions were carried out as follows: 65% ethanol (30 mL) was added to 1.25 g roots and subjected to sonication at 60 °C for 30 min in an ultrasound water bath (ultrasonic frequency 35 kHz). The extracts were filtered through Whatman filter paper, and the residues were re-extracted for two more times with 65% ethanol; the pooled filtrates were evaporated to dryness under vacuum at <40 °C and the dried extracts were stored at −20 °C until further use.

### 4.2. Phytochemical Composition

Total phenolic content (TPC) and total phenolic acid content (TPAC) were determined according to previously described methods [26,27]. Briefly, TPC was determined by using a Folin–Ciocalteu reagent and the extracts (50 µL, 1 mg/mL) was firstly mixed with the diluted reagent (100 µL; 1:9, *v*/*v*). After three minutes, sodium carbonate (75 µL, 2%) was added and the mixture was incubated in the dark for 2 h. The absorbance was measured at 765 nm. TPC was expressed as mg gallic acid equivalents (GAE)/g d.w. root. TPAC in the tested extracts was determined by Arnow’s method. The plant extracts (50 µL, 1 mg/mL) were mixed with Arnow’s reagents including sodium nitrite and sodium molybdate. Then, 50 µL of hydrochloric acid (0.5 M) was added, and 100 µL of sodium hydroxide was added. After 10 min, the absorbance was recorded at 490 nm. TPAC was expressed as mg caffeic acid equivalents (CAE)/g d.w. root. The quantitative analysis of major phenolic markers (rosmarinic acid, globoidnan, globoidnan B, and rabdosiin) was carried by HPLC-DAD on a Shimadzu system (Tokyo, Japan) after a method previously detailed in [14]. The quantitative analysis of pyrrolizidine alkaloids (intermedine, lyocopsamine, acetylintermedine/acetyllycopsamine, intermedine-*N*-oxide, lycopsamine-*N*-oxide, acetylintermedine-*N*-oxide/acetyllycopsamine-*N*-oxide) was performed by HPLC-MS/MS on an Agilent 1260 Infinity HPLC system (PaloAlto, CA, USA) coupled with a QTRAP4500 triple quadrupole MS (AB Sciex Intrusments, Framingham, MA, USA) following a previously reported analytical method [7]. The content of individual phenolic pyrrolizidine alkaloidal markers was expressed as mg/g d.w. root.

### 4.3. Antioxidant and Enzyme Inhibitory Assays

1,1-Diphenyl-2-picrylhydrazyl (DPPH) and 2,2′-azino-bis(3-ethylbenzothiazoline) 6-sulfonic acid (ABTS) radical scavenging, cupric ion reducing antioxidant capacity (CUPRAC), ferric ion reducing antioxidant power (FRAP), metal chelating ability (MCA), and phosphomolybdenum assay (PDA) were performed according to methods previously described in [26]. The antioxidant potential was expressed as mg Trolox equivalents (TE)/g d.w. root in DPPH, ABTS, CUPRAC, and FRAP assays, mg EDTA equivalents (EDTAE)/g d.w. root in MCA, and mmol TE/g d.w. root in PDA. The acetylcholinesterase (AChE), butyrylcholinesterase (BChE), tyrosinase, amylase, and glucosidase assays were performed as detailed in [26,27]. The anti-enzymatic activities were expressed as mg galanthamine equivalents (GALAE)/g d.w. root in AChE and BChE assays, mg kojic acid equivalents (KAE)/g d.w. root in tyrosinase assay, and mmol acarbose equivalents (ACAE)/g d.w. root in amylase and glucosidase assays.

### 4.4. Statistical Analysis

All the phytochemical analyses and biological assays were performed in triplicate and the results were reported as mean ± standard deviation (SD). One-way analysis of variance with Turkey’s post-hoc test (*p* < 0.05) was conducted using OriginPro2020 (OriginLab Corp., Northampton, USA). The principal component analysis (PCA) was performed after Pareto standardization of the data, whereas cluster image map (CIM) analysis was based on the Euclidean distance and Ward’s rule. The correlation analysis between the phytochemical composition and biological activities was considered significant for Pearson’s coefficients > 0.7. PCA, and CIM and analyses were conducted using R software (v. 3.6.2).

## 5. Conclusions

In the current study, the influence of the post-harvest storage time on the chemico-biological potential of comfrey roots collected from different European regions was investigated for the first time. The levels of total phenolic, total phenolic acids, individual phenolic (i.e., rosmarinic acid, globoidnan A, globoidnan B, and rabdosiin) and pyrrolizidine alkaloidal markers showed a high inter- and intra-sample variability that could be linked to the geographical collection site and post-harvest storage period. In contrast, the antioxidant and anti-enzymatic properties did not show straightforward changes that could be easily connected in a spatio-temporal manner. In conclusion, our study brings new insights into the shelf life of comfrey-based cosmeceutical and pharmaceutical preparations that could improve their industrial exploitation.

## Figures and Tables

**Table 1:** Quantitative assessment of total phenolics, total phenolic acids and individual phenolic markers in comfrey (*S. officinale*) roots collected from different European regions and stored for 1–6 months.

Storage Period (Months)	TPC	TPAC	Rosmarinic Acid	Globoidnan A	Globoidnan B	Rabdosiin
	mg GAE/g d.w. Root	mg CAE/g d.w. Root	mg/g d.w. root			
*Sample FS*						
M1	9.24 ± 0.01 ^a^	3.70 ± 0.09 ^a^	3.09 ± 0.05 ^a^	2.29 ± 0.10 ^a^	1.60 ± 0.07 ^a^	0.38 ± 0.01 ^a^
M2	8.40 ± 0.17 ^b^	5.05 ± 0.17 ^b^	1.89 ± 0.04 ^b^	1.87 ± 0.06 ^a^	0.96 ± 0.04 ^b^	0.28 ± 0.01 ^b^
M3	11.12 ± 0.21 ^c^	4.94 ± 0.18 ^b^	2.63 ± 0.07 ^c^	3.15 ± 0.26 ^b^	0.80 ± 0.07 ^b^	0.38 ± 0.02 ^a^
M6	6.48 ± 0.08 ^d^	2.67 ± 0.19 ^c^	1.65 ± 0.03 ^b^	1.24 ± 0.13 ^c^	0.80 ± 0.07 ^b^	0.14 ± 0.02 ^c^
*Sample IS*						
M1	8.67 ± 0.21 ^b^	2.87 ± 0.13 ^c^	1.81 ± 0.06 ^b^	3.17 ± 0.18 ^b^	1.50 ± 0.10 ^a^	0.52 ± 0.03 ^d^
M2	10.30 ± 0.02 ^c^	4.15 ± 0.57 ^d^	1.40 ± 0.01 ^c^	3.20 ± 0.08 ^b^	0.65 ± 0.03 ^c^	0.41 ± 0.05 ^a^
M3	11.08 ± 0.17 ^c^	5.11 ± 0.31 ^b^	1.41 ± 0.04 ^c^	2.43 ± 0.05 ^a^	0.87 ± 0.02 ^b^	0.39 ± 0.01 ^a^
M6	10.25 ± 0.21 ^c^	4.34 ± 0.31 ^b,d^	1.37 ± 0.07 ^c^	1.99 ± 0.17 ^a^	1.12 ± 0.07 ^d^	0.30 ± 0.01 ^b^
*Sample NT*						
M1	16.57 ± 0.15 ^e^	9.03 ± 0.16 ^e^	4.51 ± 0.22 ^d^	3.34 ± 0.33 ^b^	0.50 ± 0.01 ^c^	0.85 ± 0.07 ^e^
M2	12.85 ± 0.13 ^c^	5.73 ± 0.08 ^f^	3.60 ± 0.35 ^e^	3.60 ± 0.35 ^b^	1.05 ± 0.09 ^d^	0.87 ± 0.11 ^e^
M3	15.47 ± 0.17 ^e^	6.54 ± 0.32 ^g^	5.05 ± 0.12 ^f^	2.55 ± 0.22 ^a^	0.86 ± 0.08 ^b^	0.88 ± 0.08 ^e^
M6	8.68 ± 0.04 ^b^	3.74 ± 0.25 ^a^	2.82 ± 0.05 ^a^	1.37 ± 0.08 ^c^	0.57 ± 0.01 ^c^	0.42 ± 0.02 ^a^
*Sample CH*						
M1	8.42 ± 0.01 ^b^	4.07 ± 0.13 ^b^	4.98 ± 0.08 ^f^	2.33 ± 0.25 ^a^	2.50 ± 0.26 ^e^	1.26 ± 0.09 ^f^
M2	6.85 ± 0.05 ^d^	3.00 ± 0.14 ^c^	4.26 ± 0.12 ^d^	3.27 ± 0.11 ^b^	1.25 ± 0.04 ^d^	0.97 ± 0.04 ^e^
M3	10.27 ± 0.21 ^c^	4.82 ± 0.28 ^b^	4.22 ± 0.17 ^d^	2.54 ± 0.53 ^a^	1.66 ± 0.30 ^a^	1.03 ± 0.19 ^e^
M6	7.65 ± 0.28 ^f^	3.67 ± 0.01 ^a^	3.50 ± 0.09 ^e^	1.59 ± 0.05 ^c^	1.53 ± 0.02 ^a^	0.70 ± 0.04 ^g^
*Sample PL*						
M1	8.61 ± 0.05 ^b^	3.43 ± 0.27 ^a^	1.74 ± 0.02 ^b^	1.11 ± 0.03 ^c^	0.47 ± 0.02 ^c^	0.90 ± 0.05 ^e^
M2	6.95 ±0.17 ^d^	2.91 ± 0.10 ^c^	1.26 ± 0.07 ^c^	0.78 ± 0.03 ^d^	0.31 ± 0.01 ^f^	0.57 ± 0.06 ^d^
M3	9.63 ± 0.10 ^g^	4.27 ± 0.37 ^d^	1.66 ± 0.14 ^b^	0.97 ± 0.15 ^d^	0.33 ± 0.06 ^f^	0.70 ± 0.15 ^d,e^
M6	10.55 ± 0.13 ^c^	4.26 ± 0.11 ^d^	1.23 ± 0.05 ^c^	0.90 ± 0.05 ^c,d^	0.43 ± 0.02 ^c^	0.64 ± 0.03 ^d^

Data are presented as mean ± standard deviation (SD) of three determinations; values sharing different superscripts within columns are significantly different at *p* < 0.05 (Tukey’s test); CAE, caffeic acid equivalents; GAE, gallic acid equivalents; TPAC; total phenolic acid content; TPC, total phenolic content.

**Table 2:** Quantitative assessment of pyrrolizidine alkaloids in comfrey (*S. officinale*) roots collected from different European regions and stored for 1–6 months.

Storage Period (Months)	Intermedine	Lycopsamine	Acetylintermedine+ Acetyllycopsamine *	Intermedine- *N*-Oxide	Lycopsamine- *N*-Oxide	Acetylintermedine-*N*-Oxide+ Acetyllycopsamine-*N*-Oxide *
	mg/g d.w. Root					
*Sample FS*						
M1	Nq	Nq	0.03 ± 0.00 ^a^	0.14 ± 0.01 ^a^	0.50 ± 0.02 ^a^	4.26 ± 0.09 ^a^
M2	Nq	Nq	0.03 ± 0.01 ^a,c^	0.12 ± 0.01 ^a^	0.40 ± 0.06 ^b^	3.50 ± 0.66 ^b^
M3	Nq	Nq	0.01 ± 0.00 ^b^	0.01 ± 0.00 ^b^	0.36 ± 0.02 ^b^	2.78 ± 0.05 ^c^
M6	Nq	Nq	0.01 ± 0.00 ^b^	0.01 ± 0.00 ^b^	0.22 ± 0.01 ^c^	1.67 ± 0.03 ^d^
*Sample IS*						
M1	Nq	Nq	0.02 ± 0.00 ^b,c^	0.36 ± 0.02 ^c^	0.18 ± 0.01 ^c,d^	2.21 ± 0.10 ^e^
M2	Nq	Nq	0.01 ± 0.00 ^b^	0.29 ± 0.01 ^d^	0.15 ± 0.01 ^d,e^	1.78 ± 0.01 ^d^
M3	Nq	Nq	0.01 ± 0.00 ^b^	0.22 ± 0.01 ^e^	0.11 ± 0.01 ^e^	1.30 ± 0.02 ^f^
M6	Nq	Nq	0.01 ± 0.00 ^b^	0.23 ± 0.00 ^e^	0.11 ± 0.01 ^e^	1.39 ± 0.06 ^f^
*Sample NT*						
M1	Nq	Nq	0.03 ± 0.00 ^a^	0.08 ± 0.00 ^f^	0.28 ± 0.00 ^f^	1.03 ± 0.03 ^f^
M2	Nq	Nq	0.02 ± 0.00 ^b,c^	0.07 ± 0.00 ^g^	0.21 ± 0.01 ^c^	0.87 ± 0.03 ^g^
M3	Nq	Nq	0.01 ± 0.00 ^b^	0.07 ± 0.00 ^g^	0.21 ± 0.01 ^c^	0.85 ± 0.02 ^g^
M6	Nq	Nq	0.01 ± 0.00 ^b^	0.04 ± 0.00 ^g^	0.11 ± 0.01 ^e^	0.49 ± 0.02 ^h^
*Sample CH*						
M1	0.01 ± 0.00 ^a^	0.01 ± 0.00 ^a^	0.14 ± 0.00 ^d^	0.18 ± 0.00 ^h^	0.25 ± 0.01 ^c,f^	3.08 ± 0.08 ^b^
M2	0.01 ± 0.00 ^a^	0.01 ± 0.00 ^a^	0.10 ± 0.01 ^e^	0.14 ± 0.01 ^a^	0.21 ± 0.01 ^c^	2.56 ± 0.08 ^c^
M3	0.01 ± 0.00 ^a^	0.01 ± 0.00 ^a^	0.09 ± 0.01 ^f^	0.12 ± 0.00 ^a^	0.15 ± 0.00 ^d^	1.90 ± 0.11 ^d^
M6	0.01 ± 0.00 ^a^	0.01 ± 0.00 ^a^	0.08 ± 0.02 ^f^	0.13 ± 0.03 ^a^	0.20 ± 0.05 ^c^	1.83 ± 0.61 ^d^
*Sample PL*						
M1	Nq	Nq	0.02 ± 0.00 ^b,c^	0.22 ± 0.00 ^e^	0.19 ± 0.01 ^c^	2.43 ± 0.08 ^c^
M2	Nq	Nq	0.02 ± 0.00 ^b,c^	0.17 ± 0.01 ^h^	0.15 ± 0.01 ^d^	1.83 ± 0.03 ^d^
M3	Nq	Nq	0.02 ± 0.00 ^b,c^	0.18 ± 0.01 ^h^	0.14 ± 0.01 ^d^	1.81 ± 0.08 ^d^
M6	Nq	Nq	0.02 ± 0.00 ^b,c^	0.19 ± 0.01 ^h^	0.17 ± 0.02 ^c^	1.45 ± 0.07 ^f^

Data are presented as mean ± standard deviation (SD) of three determinations; values sharing different superscripts within columns are significantly different at *p* < 0.05 (Tukey’s test); Nq, not quantified; * quantified as sum of stereoisomers.

**Table 3:** Assessment of the antioxidant activity of comfrey (*S. officinale*) roots collected from different European regions and stored for 1–6 months.

Storage Period (Months)	DPPH	ABTS	CUPRAC	FRAP	MCA	PDA
	mg TE/g d.w. Root				mg EDTAE/g d.w. Root	mmol TE/g d.w. Root
*Sample FS*						
M1	5.36 ± 0.17 ^a^	34.95 ± 0.14 ^a^	13.25 ± 0.13 ^a^	13.85 ± 0.76 ^a^	2.80 ± 0.07 ^a^	0.25 ± 0.03 ^a^
M2	16.60 ± 0.07 ^b^	37.26 ± 0.74 ^b^	39.80 ± 0.55 ^b^	40.91 ± 1.27 ^b^	3.19 ± 0.12 ^b^	0.36 ±0.00 ^b^
M3	26.59 ± 0.27 ^c^	100.72 ± 4.21 ^c^	52.05 ± 0.29 ^c^	55.38 ± 1.12 ^c^	4.52 ± 0.22 ^c^	0.56 ± 0.02 ^c^
M6	16.55 ± 0.17 ^b^	71.49 ± 0.94 ^d^	32.39 ± 0.68 ^d^	34.14 ± 0.80 ^d^	2.62 ± 0.17 ^a^	0.27 ± 0.01 ^a^
*Sample IS*						
M1	24.05 ± 0.36 ^d^	118.87 ± 2.03 ^e^	42.48 ± 0.30 ^e^	42.68 ± 0.04 ^b^	7.61 ± 0.35 ^d^	0.48 ± 0.03 ^d^
M2	28.21 ± 0.70 ^e^	123.03 ± 7.51 ^f^	50.21 ± 1.98 ^c^	52.14 ± 0.32 ^e^	6.73 ± 0.30 ^e^	0.69 ± 0.02 ^e^
M3	26.92 ± 0.58 ^c^	130.95 ± 0.72 ^g^	60.74 ± 1.37 ^f^	64.93 ± 1.02 ^f^	6.34 ± 0.35 ^e,f^	0.59 ± 0.02 ^c^
M6	24.13 ± 0.42 ^d^	122.14 ± 1.61 ^f^	52.67 ± 0.71 ^c^	52.63 ± 0.85 ^c^	5.90 ± 0.46 ^f^	0.61 ± 0.01 ^c^
*Sample NT*						
M1	38.15 ± 0.32 ^f^	157.76 ± 5.54 ^h^	80.13 ± 0.87 ^g^	90.67 ± 1.74 ^g^	2.59 ± 0.18 ^a^	0.79 ± 0.02 ^f^
M2	26.78 ± 0.21 ^c^	129.12 ± 0.68 ^g^	62.28 ± 1.72 ^f^	70.62 ± 0.23 ^h^	3.69 ± 0.24 ^g^	0.53 ± 0.01 ^g^
M3	38.49 ± 0.41 ^f^	173.25 ± 1.43 ^i^	76.36 ± 0.97 ^h^	86.91 ± 1.10 ^f^	3.37 ± 0.32 ^g^	0.85 ± 0.03 ^h^
M6	21.28 ± 0.34 ^g^	83.81 ± 1.73 ^j^	39.19 ± 0.97 ^b^	48.02 ± 0.63 ^i^	1.91 ± 0.08 ^h^	0.48 ± 0.01 ^d^
*Sample CH*						
M1	8.19 ± 0.26 ^h^	53.45 ± 0.21 ^k^	20.27 ± 0.20 ^i^	21.18 ± 1.16 ^j^	4.28 ± 0.10 ^i^	0.39 ± 0.04 ^b^
M2	16.48 ± 0.44 ^b^	92.71 ± 1.02 ^l^	29.39 ± 0.91 ^d^	38.58 ± 0.17 ^k^	4.65 ± 0.22 ^c^	0.44 ± 0.03 ^d^
M3	29.25 ± 0.23 ^e^	96.37 ± 2.70 ^m^	46.85 ± 0.48 ^j^	51.93 ± 1.23 ^c^	7.43 ± 0.06 ^d^	0.50 ± 0.01 ^g^
M6	2.19 ± 0.45 ^i^	10.02 ± 2.41 ^n^	8.66 ± 0.07 ^i^	8.24 ± 0.10 ^l^	0.54 ± 0.04 ^j^	0.15 ± 0.02 ^i^
*Sample PL*						
M1	25.20 ± 1.22 ^c,d^	114.09 ± 0.18 ^e^	41.75 ± 0.69 ^e^	45.78 ± 0.49 ^i^	5.38 ± 0.66 ^f^	0.72 ± 0.01 ^e^
M2	17.99 ± 0.24 ^b^	74.08 ± 1.70 ^d^	29.89 ± 0.07 ^d^	36.75 ± 0.12 ^d,k^	3.77 ± 0.42 ^g^	0.58 ± 0.03 ^c^
M3	24.04 ± 0.89 ^d^	106.90 ± 5.13 ^c^	42.61 ± 0.89 ^e^	51.48 ± 0.40 ^c^	4.15 ± 0.64 ^c^	0.81 ± 0.04 ^f^
M6	27.44 ± 0.40 ^c^	128.22 ± 2.62 ^g^	43.17 ± 1.14 ^e^	56.00 ± 0.46 ^c^	5.20 ± 0.31 ^f^	0.79 ± 0.02 ^f^

Data are presented as mean ± standard deviation (SD) of three determinations; values sharing different superscripts within columns are significantly different at *p* < 0.05 (Tukey’s test); ABTS, 2,2′-azino-bis(3-ethylbenzothiazoline) 6-sulfonic acid; CUPRAC, cupric ion reducing antioxidant capacity; DPPH, 1,1-diphenyl-2-picrylhydrazyl; EDTAE, EDTA equivalents; FRAP, ferric ion reducing antioxidant power; MCA, metal chelating activity; PDA, phosphomolybdenum activity; TE, trolox equivalents.

**Table 4:** Assessment of the enzyme inhibitory activity of comfrey (*S. officinale*) roots collected from different European regions and stored for 1–6 months.

Storage Period (Months)	AChE	BChE	Tyrosinase	Amylase	Glucosidase
	mg GALAE/g d.w. Root	mg GALAE/g d.w. Root	mg KAE/g d.w. Root	mmol ACAE/g d.w. Root	
*Sample FS*					
M1	Na	0.69 ± 0.03 ^a^	3.97 ± 0.48 ^a^	0.10 ± 0.01 ^a^	0.29 ± 0.01 ^a^
M2	Na	0.49 ± 0.01 ^b^	5.30 ± 0.31 ^b^	0.04 ± 0.00 ^b,c^	0.20 ± 0.00 ^b^
M3	Na	0.85 ± 0.03 ^c^	6.92 ± 0.21 ^c^	0.05 ± 0.00 ^b^	0.33 ± 0.00 ^b^
M6	Na	0.52 ± 0.01 ^b^	4.23 ± 0.41 ^a^	0.03 ± 0.00 ^c^	0.21 ± 0.00 ^c^
*Sample IS*					
M1	Na	1.13 ± 0.03 ^d^	6.56 ± 0.11 ^d^	0.08 ± 0.01 ^a^	0.45 ± 0.00 ^d^
M2	Na	1.06 ± 0.02 ^e^	6.98 ± 0.68 ^c^	0.07 ± 0.00 ^d^	0.41 ± 0.01 ^e^
M3	Na	1.01 ± 0.05 ^f^	7.17 ± 0.66 ^c^	0.07 ± 0.00 ^d^	0.38 ± 0.01 ^f^
M6	Na	1.04 ± 0.03 ^e,f^	6.67 ± 0.16 ^d^	0.06 ± 0.01 ^b,d^	0.41 ± 0.01 ^e^
*Sample NT*					
M1	3.97 ± 0.01 ^a^	1.03 ± 0.04 ^e,f^	14.46 ± 0.27 ^e^	0.09 ± 0.00 ^a^	0.49 ± 0.00 ^g^
M2	2.91 ± 0.01 ^b^	0.78 ± 0.01 ^g^	9.47 ± 0.24 ^f^	0.07 ± 0.00 d	0.35 ± 0.00 ^h^
M3	4.21 ± 0.03 ^c^	1.17 ± 0.04 ^d^	13.80 ± 0.61 ^e^	0.09 ± 0.00 ^a^	0.51 ± 0.01 ^i^
M6	2.23 ± 0.02 ^d^	0.66 ± 0.01 ^a^	6.28 ± 0.58 ^d^	0.04 ± 0.00 ^b^	0.27 ± 0.00 ^a^
*Sample CH*					
M1	Na	1.05 ± 0.05 ^e^	6.06 ± 0.73 ^d^	0.16 ± 0.00 ^e^	0.44 ± 0.01 ^d^
M2	Na	1.04 ± 0.06 ^e^	13.63 ± 0.27 ^e^	0.12 ± 0.01 ^f^	0.45 ± 0.00 ^d^
M3	Na	1.06 ± 0.01 ^e^	8.23 ± 0.65 ^c^	0.11 ± 0.01 ^f^	0.43 ± 0.01 ^d^
M6	3.32 ± 0.04 ^e^	1.04 ± 0.08 ^e^	18.08 ± 0.19 ^g^	0.06 ± 0.00 ^d^	0.40 ± 0.00 ^e^
*Sample PL*					
M1	3.74 ± 0.00 ^f^	1.11 ± 0.00 ^d^	7.27 ± 0.32 ^c^	0.08 ± 0.00 ^a^	0.46 ± 0.00 ^d^
M2	2.67 ± 0.02 ^g^	0.77 ± 0.02 ^g^	9.14 ± 0.39 ^f^	0.06 ± 0.00 ^d^	0.32 ± 0.00 ^b^
M3	3.81 ± 0.01 ^h^	1.09 ± 0.05 ^d^	18.05 ± 0.47 ^g^	0.08 ± 0.00 ^a^	0.47 ± 0.00 ^g^
M6	4.08 ± 0.02 ^j^	1.07 ± 0.08 ^d,e^	18.01 ± 0.49 ^g^	0.09 ± 0.00 ^a^	0.51 ± 0.00 ^i^

Data are presented as mean ± standard deviation (SD) of three determinations; values sharing different superscripts within columns are significantly different at *p* < 0.05 (Tukey’s test); Na, not active; ACAE, acarbose equivalents; AChE, acetylcholinesterase; BChE, butyrylcholinesterase; GALAE, galanthamine equivalents; KAE, kojic acid equivalents.

## Supplementary Materials

The following are available online at https://www.mdpi.com/article/10.3390/plants10091825/s1, Table S1: Identification data of comfrey root samples.
