# Supplementary material for: Influence of the Post-Harvest Storage Time on the Multi-Biological Potential, Phenolic and Pyrrolizidine Alkaloid Content of Comfrey (Symphytum officinale L.) Roots Collected from Different European Regions
Source: Plants (Basel). 2021 Sep 2;10(9):1825. doi: 10.3390/plants10091825 (PMC8471851; doi:10.3390/plants10091825)
Supplement: Supplementary file 1 [file plants-10-01825-s001.zip › plants-1360874-supplementary.pdf]

Table S1

Identification data of comfrey root samples

Sample code	Country	Place	GPS	Voucher
FS	Germany	Freising	48.400335, 11.723935	SO0310/2020FS
IS	Romania	Iasi	47.187379, 27.574481	SO0210/2020IS
NT	Romania	Neamt	46.845291, 26.887946	SO2609/2020NT
CH	Switzerland	Uttwil	47.5832514, 9.3426943	SO2409/2020CH
PL	Poland	Lublin	51.2565091, 22.5624355	SO0110/2020PL
